# Dissolved Organic Matter Composition and Microbial Functional Traits Regulate Carbon Mineralization Efficiency in Peatland Soils Under Experimental Warming and Nutrient Input

**DOI:** 10.3390/microorganisms14061190

**Published:** 2026-05-25

**Authors:** Yixinfei Lin, Hongfeng Bian, Yanan Liu, Pengchen Zhou, Xue Wang

**Affiliations:** State Environmental Protection Key Laboratory of Wetland Ecology and Vegetation Restoration, School of Environment, Northeast Normal University, Changchun 130117, China; linyxf221@nenu.edu.cn (Y.L.); liuyanan0226@163.com (Y.L.); 15660956776@163.com (P.Z.); wangx881@nenu.edu.cn (X.W.)

**Keywords:** microbial functional traits, carbon mineralization efficiency, dissolved organic matter, warming, nitrogen input

## Abstract

Microbial functional traits play a central role in regulating carbon mineralization efficiency (CME) in peatlands, yet how they respond to concurrent warming and atmospheric nitrogen deposition remains unclear. In this study, peat soils from three vegetation types (sedge, reed, and shrub) were subjected to controlled microcosm incubations simulating warming and nitrogen addition gradients. Microbial community composition and functional profiles were characterized using 16S rRNA high-throughput sequencing and Functional Annotation of Prokaryotic Taxa (FAPROTAX) functional prediction, while dissolved organic matter (DOM) composition was analyzed via excitation–emission matrix fluorescence spectroscopy with parallel factor analysis (EEM-PARAFAC) and fluorescence indices. Integrating correlation analysis, Random Forest, and partial least squares path modeling (PLS-PM) modeling, we identified microbial functional traits as key factors linking environmental changes to soil CME, with DOM serving as a substrate-mediated pathway. External nitrogen input primarily drove shifts in microbial functional composition, whereas warming modulated substrate utilization preferences and DOM turnover. The interaction between warming and nitrogen selectively reshaped microbial functional profiles, thereby jointly determining CME. Functional traits explained more variation in CME than taxonomic composition, indicating a “structure–function decoupling” under environmental change. These findings highlight the central role of microbial functional traits in peatland carbon transformation and suggest that the net response of peatland carbon emissions to future environmental change will depend critically on the balance between warming magnitude and nitrogen deposition levels.

## 1. Introduction

Peatlands are a unique type of wetland ecosystem, covering only approximately 3% of the global terrestrial surface, yet storing more than one-third of the world’s soil carbon, making them among the most carbon-dense ecosystems on Earth [1,2,3]. Under conditions of long-term water saturation, low temperatures, and nutrient limitation, organic matter decomposition in peat soils is constrained, leading to the accumulation of large and relatively stable carbon pools [4,5,6]. However, ongoing global warming [1,7] and increasing nitrogen deposition [8,9] have been shown to alter peatland carbon cycling processes, potentially weakening carbon storage capacity and shifting peatlands from carbon sinks to carbon sources [10,11,12].

Climate warming and nitrogen inputs are widely recognized as key drivers of soil carbon dynamics. Warming is generally associated with increased microbial activity and accelerated organic matter decomposition, thereby enhancing carbon mineralization [13,14,15]. In contrast, nitrogen input alters nutrient availability and substrate composition, which may influence microbial resource utilization strategies and soil carbon turnover [16,17]. A growing body of empirical studies has demonstrated that warming and nitrogen addition not only modify the magnitude and direction of soil greenhouse gas fluxes [18,19,20] but also drive shifts in microbial functional traits, which in turn are associated with organic matter transformation processes [21].

Dissolved organic matter (DOM) represents an active and bioavailable fraction of soil organic carbon and serves as an important substrate for microbial processes [22,23,24,25]. Variations in DOM composition, particularly in terms of humification and molecular complexity, influence substrate availability and may be associated with shifts in microbial functional characteristics and carbon mineralization patterns [22,26,27]. Environmental changes such as warming and nitrogen input can modify both the quality and quantity of DOM, thereby influencing its potential role in soil carbon cycling [27,28].

Soil microorganisms play a central role in soil carbon cycling through their involvement in decomposition and transformation processes [29]. Beyond carbon metabolism, microorganisms mediate a range of ecosystem functions critical to peatland responses to global change. These include nitrogen transformations (e.g., nitrification and denitrification) [30], methane oxidation [31], and sulfur cycling [32]. These processes collectively regulate greenhouse gas fluxes, nutrient availability, and redox conditions in peatland ecosystems. Shifts in microbial community composition under warming and nitrogen addition may therefore have cascading effects on multiple biogeochemical cycles, potentially reinforcing or offsetting carbon-cycle feedbacks [33,34]. Microbial functional traits have been increasingly recognized as important factors associated with soil carbon dynamics [29,35,36]. Environmental changes, such as warming and nitrogen addition, influence soil carbon cycling by altering microbial metabolic functions. These shifts may reshape the coupling between carbon and nitrogen processes. In peatland and forest soils, warming has been associated with increased abundance of Actinobacteria and Firmicutes and declines in Acidobacteria [37], alongside enhanced expression of functional genes involved in labile carbon degradation [38] and nitrogen mineralization [34]. Long-term nitrogen input suppresses the relative abundance of methane-oxidizing functional groups, reducing methane emissions [39,40]. Meanwhile, nitrogen addition can enhance microbial functions related to carbohydrate-binding modules and polysaccharide lyases, thereby increasing CO_2_ emissions [41]. In peat soils, microbial communities are highly specialized due to long-term water saturation, low nutrient availability, and anaerobic conditions. Specific functional groups, such as methanotrophs, cellulose-degrading bacteria, and recalcitrant carbon decomposers, play pivotal roles in carbon transformation and storage, as well as in broader biogeochemical processes in peatland ecosystems [42,43,44]. Variations in DOM composition can selectively stimulate these microbial functional traits, thereby potentially influencing the rate and efficiency of soil carbon mineralization. Understanding how microbial functional traits respond to DOM changes under warming and nitrogen addition is crucial for predicting peatland carbon dynamics and their feedbacks to global climate [45]. High-throughput sequencing combined with functional annotation approaches, such as Functional Annotation of Prokaryotic Taxa (FAPROTAX), can be used to infer potential microbial functional characteristics related to carbon metabolism, providing a useful proxy for exploring putative functional attributes of microbial communities involved in carbon transformation [46].

Carbon mineralization is a key process influencing soil carbon storage and stability [47]. Microbial functional traits are considered important factors associated with soil carbon mineralization, potentially influencing both the rate and the apparent efficiency with which soil organic carbon is converted to CO_2_. Here, carbon mineralization efficiency (CME) is defined as the cumulative release of CO_2_-C per unit of initial soil organic carbon (SOC), serves as an integrated measure of carbon pool vulnerability to environmental forcing [48,49,50]. This metric quantifies the proportional loss of stored carbon over a given incubation period, reflecting the apparent decomposability of the substrate rather than the physiological allocation of assimilated carbon by microorganisms. CME is therefore conceptually distinct from microbial carbon use efficiency (CUE), which describes the anabolic partitioning of substrate at the cellular level. Variation in CME arises from the interplay of microbial activity, substrate availability, and soil physicochemical properties. Labile carbon fractions provide readily accessible substrates for microbial metabolism, while microbial biomass and functional diversity are associated with decomposition potential. In this context, microbial functional traits can be viewed as a conceptual link between substrate quality (DOM) and carbon mineralization outcomes (CME), integrating environmental conditions and substrate characteristics in soil carbon dynamics. By positioning microbes at the center of this framework, the present study highlights their important role in controlling peatland carbon cycling under warming and nitrogen enrichment.

Based on this framework, this study aims to examine how warming and nitrogen addition are associated with variation in peat soil carbon mineralization and to explore the potential roles of DOM composition and inferred microbial functional traits in this process. We hypothesize that (i) warming and nitrogen addition will interactively shift DOM composition toward more humified, less bioavailable fractions; (ii) these DOM shifts will correspond with changes in inferred microbial functional traits, particularly toward increased heterotrophic respiration; and (iii) microbial functional traits will serve as a key pathway statistically linking environmental drivers to CME variation. DOM composition was characterized using EEM-PARAFAC analysis, and microbial functional traits were inferred using high-throughput sequencing combined with FAPROTAX annotation. Random Forest and PLS-PM analyses were applied to identify key factors and potential pathways linking environmental change, DOM, microbial functional traits, and CME. This approach positions inferred microbial function as the crucial link between substrate availability, environmental change, and soil carbon mineralization, providing insights into the microbial regulation of peatland carbon dynamics under global change scenarios.

## 2. Materials and Methods

### 2.1. Site Descriptions and Soil Sample Collection

**Figure 1:**
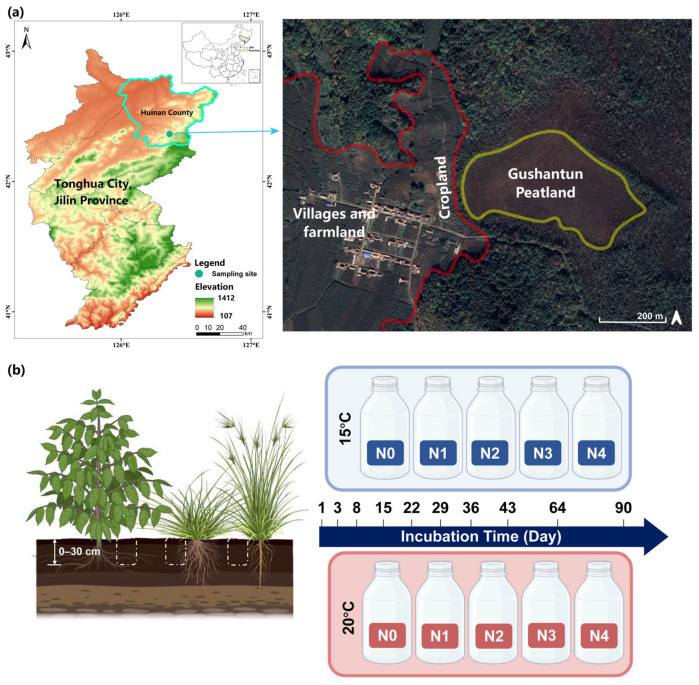
Experimental setup and sampling design. (**a**) Location of the Gushuntan peatland on the western slope of the Changbai Mountains, Northeast China. The sampling sites are marked with yellow circles within the peatland area. The red region adjacent to the peatland represents villages and cropland. (**b**) Conceptual graphs of the laboratory incubation setup. Peat soils (300 g fresh weight) from the surface soil (30 cm) of the three vegetation types were placed in 1000 mL glass bottles and incubated in the dark at 15 °C and 20 °C under five nitrogen addition levels (N0–N4: 0, 30, 60, 90, and 120 mg N kg^−1^ soil). Gas samples were collected periodically over the 90-day incubation period at the time points.

The study area is located in the Gushantun peatland on the western slope of the Changbai Mountains in Northeast China (42°18′23″ N, 126°17′32″ E; 514 m a.s.l.). The site is a peat-forming wetland that developed from a volcanic crater lake and features a basin-like topography with higher elevations surrounding a lower central area. It has undergone approximately 13,000 years of ecological succession. The dominant vegetation includes Sphagnum mosses, Phragmites australis, and Betula ovalifolia. The mean annual air temperature is approximately 5 °C, with a mean growing-season temperature of approximately 15 °C. Mean annual precipitation is about 800 mm, with the majority occurring during the summer season [51]. Since the 1960s, forested land on the southwestern side of the wetland has been converted to cropland (Figure 1a) and continuously cultivated with maize. Nitrogen fertilizer (urea) has been applied annually at a rate of approximately 230 kg·ha^−1^.

Soil samples were collected in June 2024 from peatland sites dominated by sedge (C), reed (*Phragmites australis*; LW), and shrubs (*Betula ovalifolia*; G), respectively. Within each plot, five sampling points were selected using a five-point sampling method. Surface debris, including roots, stones, and litter, was carefully removed prior to sampling. Peat soil was collected from the 0–30 cm depth using a soil auger. The five subsamples obtained from each plot were thoroughly homogenized to form one composite sample, which was then placed into self-sealing plastic bags and transported to the laboratory under refrigerated conditions (4 °C). Soil water content and bulk density were determined prior to incubation and are presented in Table S4.

### 2.2. Incubation Experiment

Based on the mean growing-season temperature of the study area and the nitrogen application rate in adjacent maize fields, a laboratory incubation experiment was designed to simulate warming and nutrient input scenarios. The experiment followed a two-factor factorial design with temperature and nitrogen addition. Urea [CO(NH_2_)_2_] was used as the nitrogen addition source, with five levels of exogenous nitrogen input: 0 (N0), 30 (N1), 60 (N2), 90 (N3), and 120 (N4) mg N kg^−1^ soil. Incubations were conducted at constant temperatures of 15 °C and 20 °C under controlled moisture conditions in the dark for 90 days. Each treatment included four independent replicates, and each incubation bottle was considered an independent experimental unit, yielding a total of 120 experimental units.

Soil moisture was maintained at 60% water-holding capacity (WHC) throughout the incubation by daily weighing and addition of distilled water. For the incubation, 300 g of fresh soil was placed into 1000 mL incubation bottles with a gas-tight lid equipped with a rubber septum and pre-incubated for 7 days under constant moisture and dark conditions at the two designated temperatures to stabilize microbial activity prior to treatment application. After the pre-incubation, urea solutions at the corresponding concentrations were added into each bottle. The N0 treatment received an equal volume of deionized water. Then all samples were incubated under dark, controlled conditions.

Gas samples were collected periodically throughout the incubation period (Figure 1b). The headspace volume of each incubation bottle was kept constant throughout the experiment: after each sampling with gas-tight syringes, the extracted volume was immediately replaced with ambient air to maintain pressure equilibrium.

After incubation, soil samples were collected and divided into three subsamples. One portion of fresh soil was stored at 4 °C for the determination of NH_4_^+^-N and NO_3_^−^-N contents. The second portion was air-dried and passed through a 100-mesh sieve for the analysis of soil physicochemical properties. The remaining portion was stored at −80 °C for microbial DNA extraction, PCR amplification, and sequencing.

### 2.3. Measurement Methods

#### 2.3.1. Measurement of Soil CO_2_ Emission Rates and CME

Soil CO_2_ emission rates were measured on days 1, 3, 8, 15, 22, 29, 36, 43, 64, and 90 following the procedures described below. On each sampling day, the headspace of each incubation bottle was flushed with ambient air for 30 min to equilibrate the headspace. The bottles were immediately sealed with gas-tight rubber stoppers after flushing. Gas samples (20 mL) were collected at 0 h and 4 h after incubation using a gas-tight syringe (50 mL) and transferred into aluminum foil gas sampling bags. The CO_2_ concentration in the gas samples was subsequently determined by gas chromatography (Agilent 7890B, Agilent Technologies, Santa Clara, CA, USA). Soil carbon emission rates were calculated using the following equation:

(1) $$\begin{array}{c} R=\frac{C\cdot M\cdot V\cdot 273}{m\cdot h\cdot 22.4\cdot \left(273+T\right)} \end{array}$$

where R is the gas emission rate (mg·kg^−1^·h^−1^); C is the difference in gas concentration between the beginning and the end of the sealed incubation (ppm); V is the headspace volume of the incubation bottle (L); m is the mass of the soil sample (kg); h is the duration of the sealed incubation (h); T is the incubation temperature (°C); and M is the molar mass of the gas, expressed on a carbon basis.

CME was calculated as cumulative CO_2_-C release per unit SOC, following previous studies in soil science [48,49].

(2) $$\begin{array}{c} CME=\frac{\sum_{i=1}^{n}\left(\left(R_{t_{i}}+R_{t_{i+1}}\right)\times \frac{t_{i+1}-t_{i}}{2}\right)}{\mathrm{SOC}} \end{array}$$

where CME represents the amount of carbon mineralized per unit of soil organic carbon; t_i_ denotes the *i*th day of incubation; R_ti_ is the gas emission rate on day i; SOC is the soil organic carbon content (g·kg^−1^).

It should be noted that CME in this study does not represent microbial CUE, but rather a relative index reflecting the extent of SOC mineralization.

#### 2.3.2. Determination of Soil Basic Parameters

Soil pH and electrical conductivity (EC) were measured in a 1:1.25 (*w*/*v*) soil-to-water suspension using the multiparameter water quality analyzer (DZB-712, INESA Scientific Instrument Co., Ltd., Shanghai, China). Soil organic carbon (SOC) content was determined using the potassium dichromate oxidation–spectrophotometric method. Soil NH_4_^+^-N and NO_3_^−^-N contents were extracted with 1 M KCl and measured using a continuous flow analyzer (SmartChem 200 Discrete Auto Analyzer, Systea, S.p.A., Anagni, Italy). The DOC and DTN were extracted with distilled water (soil: extractant = 1:10 *w*/*v*), filtered through a 0.45 μm membrane, and analyzed using a TOC analyzer (TOC-V CSH/CPN, Shimadzu, Kyoto, Japan).

#### 2.3.3. DOM Spectral Characteristics and EEM-PARAFAC Analysis

DOM samples were extracted using the soil-water shaking method [51]. Excitation-emission matrix fluorescence spectroscopy (EEM) was performed using a fluorescence spectrophotometer (F-2700, Hitachi, Tokyo, Japan). Excitation wavelengths (Ex) ranged from 250 to 500 nm and emission wavelengths (Em) from 250 to 580 nm, both at 5 nm intervals. The scanning speed was set to 3000 nm/min.

Three key optical indices were used to characterize DOM: the fluorescence index (FI), biological index (BIX), and humification index (HIX). FI and BIX are commonly associated with DOM sources, with FI values > 1.9 indicating a microbial contribution and values < 1.4 indicating a terrestrial plant contribution, while BIX values > 1.0 indicate autochthonous biological production and values between 0.6 and 0.7 indicate terrestrial input. HIX values are generally proportional to the degree of DOM humification [51,52,53].

PARAFAC modeling was performed using the drEEM toolbox [54] in MATLAB R2018a to extract five fluorescent components (hereafter referred to as CP1–CP5). These components were compared with previously reported components in the OpenFluor database (https://openfluor.lablicate.com/) [55]. In total, five fluorescent components were identified, including three humic-like components (CP1–CP3) and two microbial-metabolite-like components (CP4–CP5), as summarized in Table 1.

In general, humic-like components (CP1–CP3) are considered relatively recalcitrant and less bioavailable carbon pools [62,63], whereas protein-like components (CP5) represent more labile substrates readily utilized by microorganisms [62]. Quinone-like components (CP4), characterized by redox-active properties [64,65], may participate in electron transfer processes and influence microbial metabolic pathways.

#### 2.3.4. Soil Bacteria 16S rRNA High-Throughput Sequencing

Total genomic DNA was extracted from 0.5 g of fresh peat soil using the CTAB method. The V3–V4 hypervariable region of the bacterial 16S rRNA gene was amplified using primers 341F (5′-CCTAYGGGRBGCASCAG-3′) and 806R (5′-GGACTACNNGGGTATCTAAT-3′). PCR amplification was performed in a 15 μL reaction mixture containing 10 ng of template DNA, 0.2 μM of each primer, and Phusion High-Fidelity PCR Master Mix (New England Biolabs, Ipswich, MA, USA). Thermal cycling conditions were as follows: initial denaturation at 98 °C for 1 min; 30 cycles of 98 °C for 10 s, 50 °C for 30 s, and 72 °C for 30 s; and a final extension at 72 °C for 5 min. PCR products were examined by electrophoresis on 2% agarose gels, and target fragments were purified using magnetic bead-based purification. Purified products were quantified and pooled in equimolar concentrations. Sequencing libraries were prepared using the NEBNext Ultra II DNA Library Prep Kit (New England Biolabs, Ipswich, MA, USA), including end repair, A-tailing, adapter ligation, and purification. Library quality was verified using an Agilent 2100 Bioanalyzer and qPCR. Paired-end sequencing (2 × 250 bp) was performed on the Illumina NovaSeq 6000 platform by Novogene Bioinformatics Technology (Beijing, China), generating an average of approximately 100,000 reads per sample. All samples collected at the end of the 90-day incubation were subjected to DNA extraction and sequencing. For each treatment combination, four independent replicate samples were sequenced, yielding a total of 120 sequenced samples across the full experimental design.

#### 2.3.5. Bioinformatic Analysis of 16S rRNA Gene Sequencing Data

Raw paired-end reads were demultiplexed based on unique barcode sequences and truncated by removing barcode and primer sequences. Paired-end reads were merged using FLASH v1.2.11 with a minimum overlap of 10 bp to obtain raw tags. Quality filtering was performed using fastp v0.23.1, where low-quality bases (Phred score < 20) were trimmed and reads shorter than 50 bp after trimming were discarded, yielding high-quality clean tags. Chimera sequences were detected by comparing tags against the Silva 138.1 reference database and removed using vsearch v2.16.0 to obtain effective tags. Merged reads ranging from 400 to 500 bp were retained for subsequent analysis.

Amplicon sequence variants (ASVs) were inferred from the effective tags using the DADA2 module within QIIME2 v2022.2 with default parameters. Taxonomic assignment was performed against the Silva 138.1 reference database using a Naive Bayes classifier trained on the V3–V4 region. Sequences classified as chloroplasts, mitochondria, or unassigned at the phylum level were removed prior to downstream analysis.

To account for differences in sequencing depth, the ASV table was rarefied to the minimum sequencing depth across all samples. Alpha diversity indices, including observed ASVs, Shannon diversity and Pielou’s evenness, were calculated using QIIME2. Differences in alpha diversity among treatment groups were assessed using Kruskal–Wallis tests with Benjamini–Hochberg correction for multiple comparisons. Beta diversity was evaluated using Bray–Curtis dissimilarity and visualized via non-metric multidimensional scaling (NMDS). Statistical differences in community composition among groups were tested using PERMANOVA (Adonis) with 999 permutations.

Microbial functional profiles were inferred using the FAPROTAX database, which assigns putative ecological functions to prokaryotic taxa based on cultured representatives [66]. This approach reflects potential functional traits rather than direct measurements of gene abundance or metabolic activity.

### 2.4. Statistical Analysis

Descriptive statistics were conducted for soil physicochemical properties and fluorescence characteristics. Two-way analysis of variance (two-way ANOVA) was applied to test the effects of warming and nitrogen addition, as well as their interaction, on the measured variables. Tukey’s multiple comparison test was used for post hoc comparisons. Normality was assessed using the Shapiro–Wilk test, and homogeneity of variances was evaluated using Levene’s test. All statistical analyses were performed at a significance level of *p* < 0.05.

Spearman’s rank correlation analysis was performed to assess relationships among soil properties, DOM optical indices, microbial variables, and CME. A Random Forest model was used to evaluate the relative importance of these variables in explaining variations in CME. Finally, partial least squares path modeling (PLS-PM) was applied to infer the potential direct and indirect effects of soil properties, DOM characteristics, and microbial variables on CME. The present dataset (120 experimental units) satisfies the commonly applied “10-times rule,” which requires the sample size to be at least 10 times the number of indicators of the most complex latent variable. Model validation was performed using bootstrap resampling (1000 replicates) to estimate standard errors and 95% bias-corrected confidence intervals for all path coefficients. Paths with bootstrap confidence intervals not covering zero were considered statistically significant. The goodness-of-fit (GoF) index was used as a descriptive measure of overall model performance.

Statistical analyses were conducted using SPSS 26, QIIME2, and R 4.4.3 with the packages vegan, phyloseq, Random Forest, and plspm. Data visualization was performed using the ggplot2 package in R and the Chiplot online platform (https://www.chiplot.online/ (accessed on 1 November 2025)).

## 3. Results

### 3.1. CO_2_ Emissions and CME Under Warming and Nitrogen Addition

**Figure 2:**
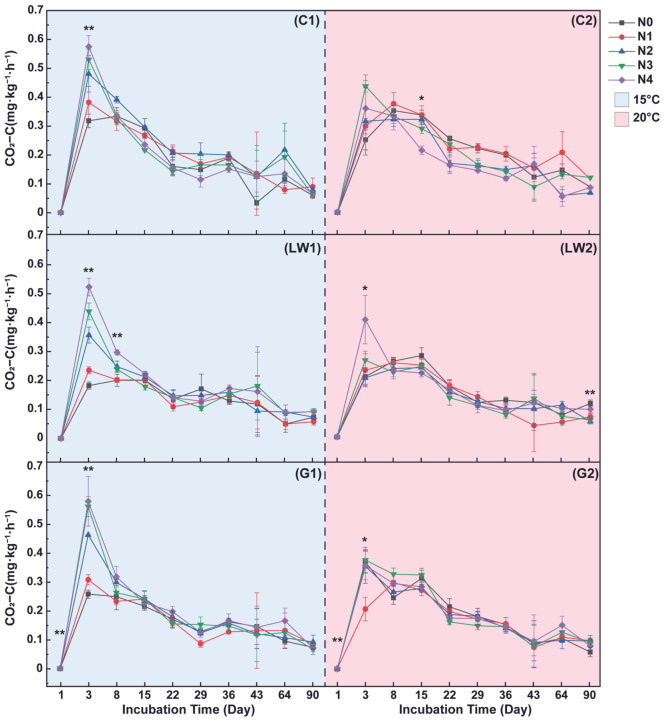
Effect of different incubation conditions on the daily CO_2_ emission rate of peatland soil. (C1, LW1, G1) daily CO_2_ emission rates of three vegetation types in peatlands at 15 °C; (C2, LW2, G2) daily CO_2_ emission rates of three vegetation types in peatlands at 20 °C. * indicates significant differences in daily emission rates (*p* < 0.05), ** indicates *p* < 0.01. Symbols represent treatment means, while error bars represent the standard error of the mean.

**Figure 3:**
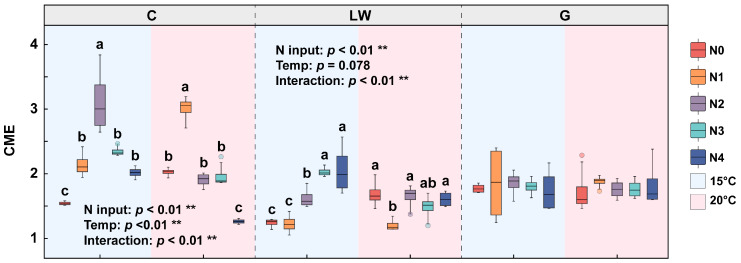
Effect of different incubation conditions on CME. Boxplot lines indicate medians with 95% confidence intervals; different lowercase letters indicate significant differences under different N inputs at *p* < 0.05. ** *p* < 0.01.

Daily soil CO_2_ emission rates during the incubation are shown in Figure 2. Across the three peatland soil types, CO_2_ emission rates increased significantly with increasing nitrogen addition (*p* < 0.05), with the highest emission observed under N4 at 15 °C. Warming to 20 °C generally delayed the emission peaks and reduced their magnitude, suggesting that it broadly attenuated the sensitivity of soil carbon emissions to exogenous nitrogen inputs. On this common background trend, CME exhibited vegetation-specific response patterns (Figure 3). Overall, in the C and LW peat soils, CME was promoted by warming under the no-N treatment (N0) (*p* < 0.01) but shifted to a significant inhibitory response under high N inputs (N3 and N4) (*p* < 0.01). This reflects a consistent directional switch in the effects of temperature and N on carbon mineralization in these two vegetation types.

However, the magnitude and pattern of responses differed among vegetation types. In the sedge peatland (C), CME exhibited a unimodal response to increasing N at both temperatures, peaking at N2 under 15 °C but shifting to an earlier peak at N1 under 20 °C. In the reed peatland (LW), CME increased approximately linearly with N at both temperatures; the main effect of temperature alone was not significant (*p* = 0.078), whereas the warming × N interaction was significant (*p* < 0.05). By contrast, CME in the shrub peatland (G) was insensitive to temperature, N addition, and their interaction (*p* > 0.05). In summary, nitrogen addition generally enhanced CO_2_ emissions and the potential for carbon mineralization across the three peatlands, while warming tended to weaken these effects; vegetation type determined the specific carbon-transformation response patterns under combined warming and N stress.

### 3.2. DOM Content and Composition Under Warming and Nutrient Input

Different vegetation types had distinct baseline soil properties and DOM composition (Tables S1 and S2). These inherent differences shaped the initial quality and microbial bioavailability of soil DOM, thereby setting the stage for the differential responses of the three peat soils to warming and nitrogen addition observed later.

**Figure 4:**
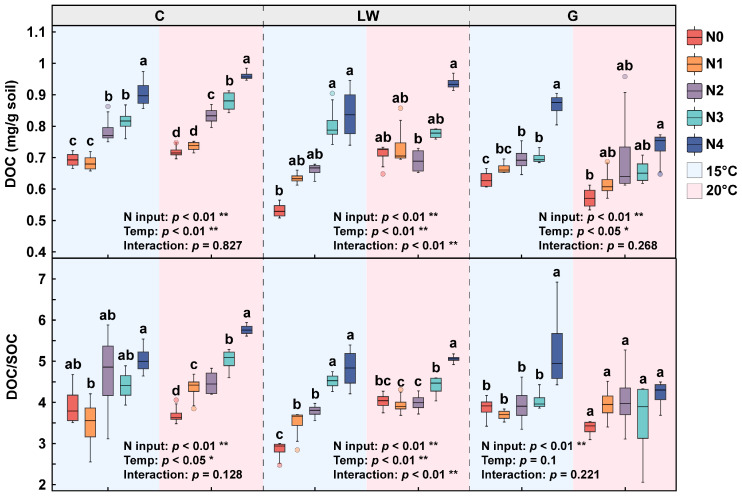
Effect of different incubation conditions on the content of DOC and DOC/SOC ratio. Different lowercase letters indicate significant differences among treatment groups within the same stage of succession at *p* < 0.05. * *p* < 0.05; ** *p* < 0.01.

The incubation experiment showed that soil DOC content and its proportion relative to SOC (DOC/SOC) differed significantly among temperature and nitrogen addition treatments (Figure 4). Overall, nitrogen addition significantly increased both parameters (*p* < 0.05), with the most pronounced increases occurring under the high nitrogen treatments (N3 and N4). Warming effects on DOC accumulation were vegetation-specific, showing significant promotion in the C and LW peat soils but only a non-significant increase in the G peat soil (*p* = 0.10). Furthermore, two-way ANOVA indicated that both nitrogen addition and warming exerted significant main effects on DOC content and DOC/SOC (*p* < 0.05), while their interaction was significant only in the LW peat soil.

**Figure 5:**
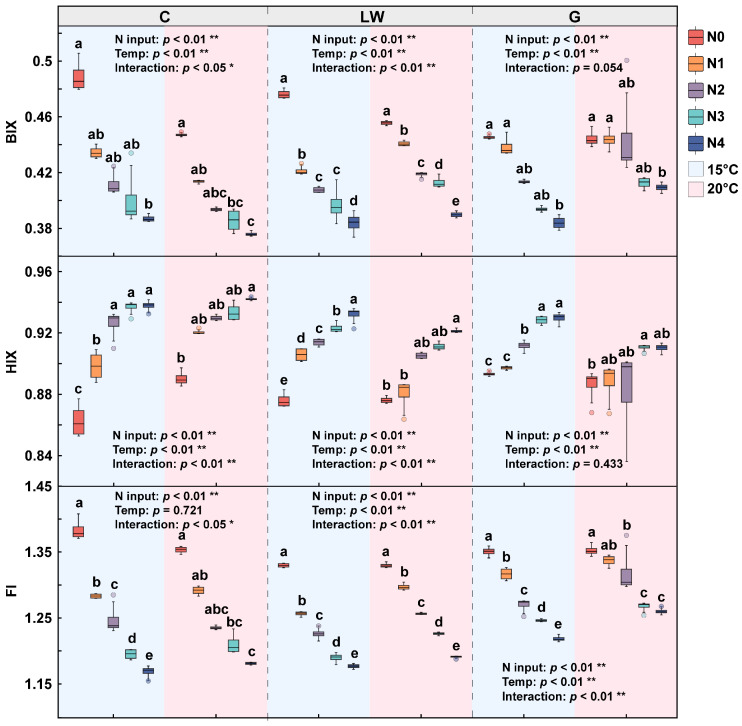
Effect of different incubation conditions on the fluorescence indices (BIX: biological index; HIX: humification index; FI: fluorescence index). Different lowercase letters indicate significant differences among treatment groups within the same stage of succession at *p* < 0.05. * *p* < 0.05; ** *p* < 0.01.

Analysis of three fluorescence indices (BIX, HIX, FI; Figure 5) demonstrated that nitrogen addition significantly drove DOM toward greater humification, decreasing BIX and FI but increasing HIX (*p* < 0.05). Warming enhanced this directional shift. Notably, the interactive effect of warming and nitrogen was significant for all indices in the C and LW soils but only affected FI in the G soil.

**Figure 6:**
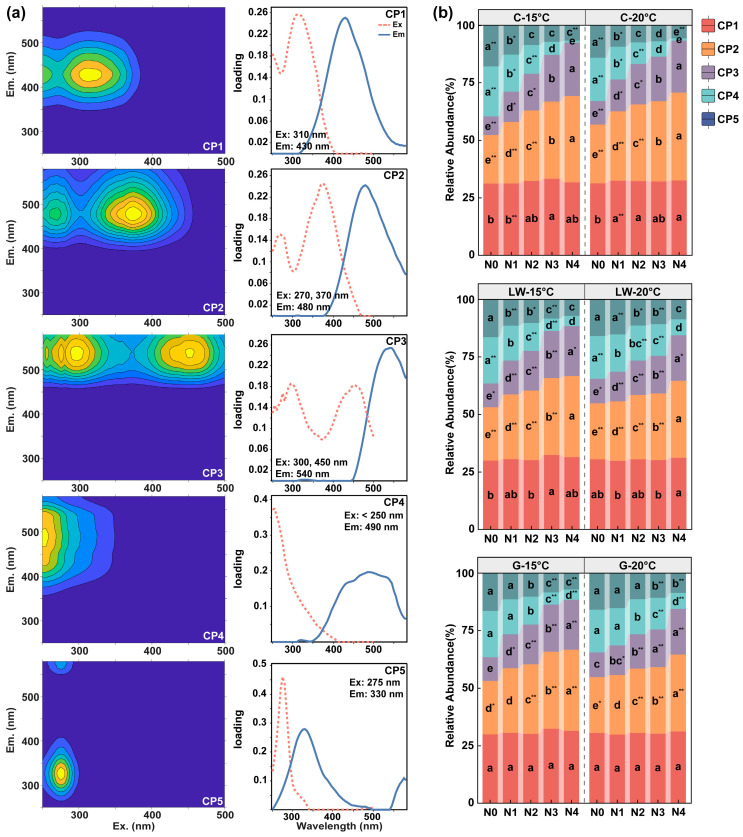
(**a**) Loadings for CP1-CP5 from the PARAFAC model, and (**b**) effect of different incubation conditions on the content of CP1–CP5. Different lowercase letters indicated significant differences at the same temperature under different N inputs at *p* < 0.05. * indicated significant differences under the same N input at the different temperature, * represents *p* < 0.05, ** represents *p* < 0.01.

PARAFAC identified five fluorescent components (Figure 6a). In response to nitrogen addition, the relative abundances of CP2 and CP3 increased, while those of CP4 and CP5 decreased significantly; CP1 remained stable (*p* > 0.05). Warming moderated these shifts, reducing the accumulation of CP2 and CP3 and mitigating the decline of CP4 and CP5. These shifts indicate a transition of DOM composition toward more humified and less bioavailable carbon fractions, accompanied by a reduction in labile substrates available for microbial utilization. The decline in CP5 suggests a depletion of easily degradable substrates, whereas changes in CP4 may reflect altered redox-active processes influencing microbial metabolic potential.

### 3.3. Microbial Community Structure and Functional Traits Under Warming and Nutrient Input

The dominant bacterial phyla differed among peatland types. In the C and G peat soils, *Proteobacteria*, *Acidobacteriota*, and *Bacteroidota* were most abundant, whereas the LW peat soil was dominated by *Proteobacteria*, *Acidobacteriota*, and *Chloroflexi* (Figure S1). For α-diversity (Shannon and Pielou’s evenness indices; Figure S2a), responses to nitrogen addition varied. A unimodal pattern was observed in C and LW peat soils, with an initial increase then decline under high N. Conversely, the G peat soil showed only a marginal, non-significant increase (*p* > 0.05). To visualize changes in bacterial community structure, non-metric multidimensional scaling (NMDS) was performed based on Bray–Curtis distances (Figure S2(b1–b4)). Community separation was driven primarily by temperature, as evidenced by clear segregation along the NMDS2 axis. Nitrogen input, however, exerted only a weak and non-significant influence. However, the lack of a significant nitrogen effect on community structure does not necessarily imply the absence of functional responses, as microbial functional traits may shift without substantial changes in taxonomic composition.

**Figure 7:**
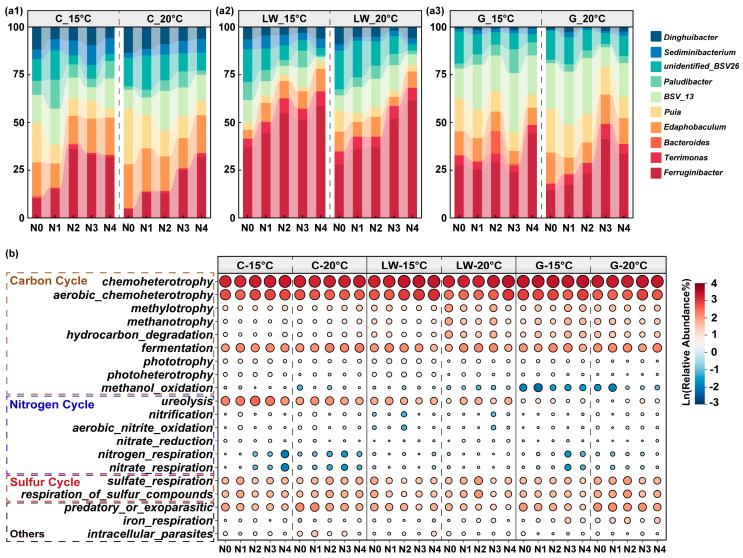
(**a1**–**a3**) Distribution of the dominant *Bacteroidota* genera (top 10) under different incubation conditions and (**b**) the heat map of the bacterial function predictions based on the FAPROTAX tool. Circle size represents relative abundance, and color represents scaled relative abundance.

Bacterial community composition was broadly stable at the phylum level, suggesting that major bacterial lineages exhibited a degree of resistance to warming and nitrogen addition. At the genus level, the three peat types harbored distinct dominant genera and exhibited divergent responses to nitrogen addition and warming (Figure S3). In sedge peat (C), the dominant genera were *Pseudolabrys*, *Massilia*, and *Bradyrhizobium*. Warming increased the relative abundance of *Pseudolabrys* but reduced that of *Bradyrhizobium*. The abundance of *GOUTA6* decreased with increasing nitrogen input, whereas *Citrifermentans* showed a modest increase under nitrogen addition. *Pseudomonas* was more abundant under ambient temperature than under warming. In reed peat (LW), the dominant genera were *Pseudolabrys, Ferruginibacter,* and *Candidatus Solibacter.* The relative abundances of *Candidatus Solibacter* and *Citrifermentans* increased initially at low-to-moderate nitrogen levels but declined under high nitrogen input (N4). *Bradyrhizobium* was less abundant under warming than at ambient temperature. In shrub peat (G), the dominant genera were *Pseudolabrys*, *GOUTA6*, and *Candidatus Solibacter*, all of which remained relatively stable under the combined warming–nitrogen treatments, indicating potential resilience of these dominant genera to environmental perturbations. *Citrifermentans* displayed a unimodal response to nitrogen addition, increasing at intermediate levels before declining at N4. Other genera showed no pronounced response to warming. Notably, only *Bacteroidota* showed a significant decrease in relative abundance with nutrient addition (*p* < 0.05). Analysis of its dominant genera (Figure 7(a1–a3)) revealed distinct responses: *Ferruginibacter* increased with nitrogen input in all peatlands; *Puia* was highly sensitive to the combined treatment, its abundance declining markedly with high nitrogen, an effect intensified by warming (*p* < 0.05). Meanwhile, *Sediminibacterium* abundance was significantly reduced by warming in the C and LW peat soils but unaffected in the G peat soil. Linear discriminant analysis effect size (LEfSe) analysis was performed to identify bacterial taxa significantly enriched under different warming and nitrogen addition treatments (LDA score > 4, *p* < 0.05) (Figure S4). Overall, low nitrogen treatments significantly enriched Acidobacteria (phylum) and Acidobacteriae (class), indicating that oligotrophic taxa adapted to low-resource environments dominated under relatively nutrient-limited conditions. In contrast, high nitrogen treatments significantly enriched Bacteroidia (class), Chitinophagales (order), Desulfuromonadia (class), and Geobacterales (order). These taxa are typically associated with the degradation of complex substrates and play key roles in regulating soil carbon and nitrogen cycling. Compared with nitrogen addition, temperature-driven enrichment of specific bacterial taxa showed community-level dependence, with the magnitude and direction of these effects varying across vegetation types and successional stages.

Bacterial ASVs were annotated to 20 functional guilds (FAPROTAX) covering C, N, and S cycle processes. Most guilds responded more strongly to temperature than to nitrogen (Figure 7b). Carbon-cycle guilds (*chemoheterotrophy*, *aerobic chemoheterotrophy*, *phototrophy*, *photoheterotrophy*) consistently decreased under warming in all peat soils. Only *chemoheterotrophy* and *aerobic chemoheterotrophy* increased significantly with nitrogen addition. Nitrogen cycle guilds (e.g., *ureolysis*, *nitrate reduction*, *nitrogen respiration*) responded similarly in C and LW peat soils, with abundances strongly reduced by warming. By contrast, sulfur cycle guilds were suppressed by warming only in the C peat soil. In the G peat soil, methane-cycle guilds (*methylotrophy*, *methanotrophy*, *hydrocarbon degradation*) were highly sensitive to the warming and nitrogen interaction. Their abundances increased significantly with nitrogen input (*p* < 0.05), with the greatest increases at low temperatures. Warming significantly weakened this stimulatory effect (*p* < 0.05). In addition to the responsive guilds, several functional groups showed no significant variation across treatments, suggesting the presence of functionally resilient components that may help maintain baseline ecosystem processes under environmental change.

### 3.4. Key Factors and Potential Pathways Associated with CME

**Figure 8:**
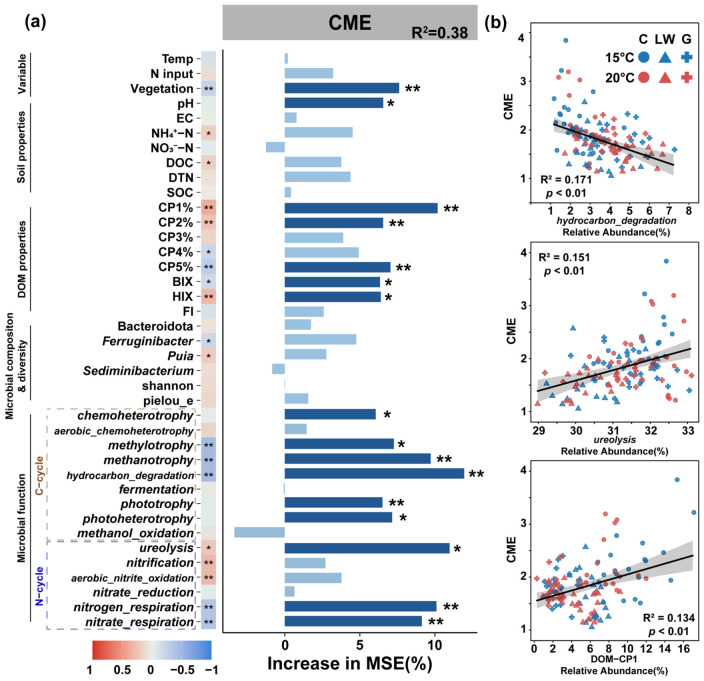
(**a**) Correlative and predictive analyses (heatmap and Random Forest) of CME with soil, DOM, and microbial variables. (**b**) Linear regression between CME and the key predictors. * represents *p* < 0.05, ** represents *p* < 0.01. Blue and red colors indicate 15 °C and 20 °C treatments, respectively, while symbols represent different vegetation types (C: sedge, LW: reed, G: shrub).

Spearman correlation and Random Forest modeling were used to identify key factors associated with CME variation (Figure 8a). CME showed significant positive correlations with NH_4_^+^–N, DOC, HIX, CP1, CP2, and the relative abundances of *Puia*, *ureolysis*, *nitrification*, and *aerobic nitrite oxidation* (*p* < 0.05). In contrast, it was negatively correlated with BIX, CP4, CP5, and the relative abundances of *Ferruginibacter*, *methylotrophy*, *methanotrophy*, *hydrocarbon degradation*, *nitrogen respiration,* and *nitrate respiration* (*p* < 0.05). To further explore variables associated with variation in CME under warming and nitrogen addition, a Random Forest analysis was performed. Model results revealed that *hydrocarbon degradation*, *ureolysis*, CP1, *nitrogen respiration*, and *methanotrophy* were identified as the most important predictors associated with CME variation. Notably, subsequent linear regression analyses showed significant associations with CME (*p* < 0.05) and explained a substantial proportion of its variance (Figure 8b).

**Figure 9:**
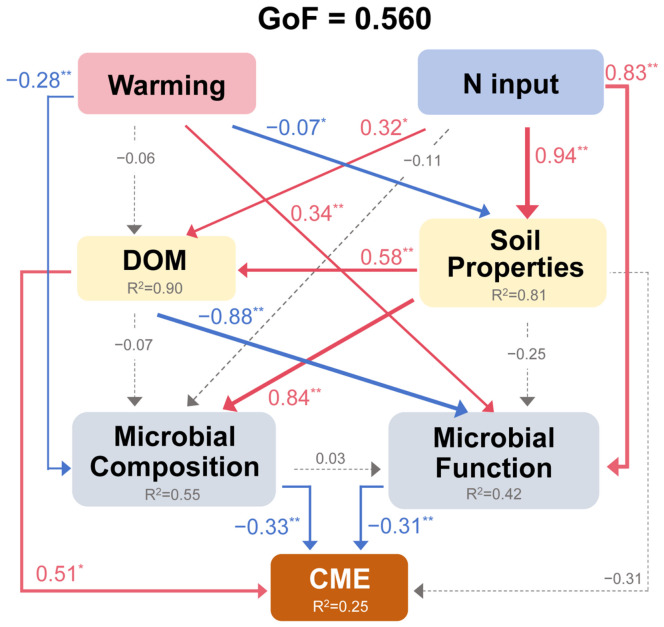
PLS-PM showing the drivers of CME via environmental factors, DOM, and microbial traits. Blue arrows indicate positive effects and red arrows indicate negative effects (* *p* < 0.05, ** *p* < 0.01); gray dashed arrows indicate non-significant paths. Numbers adjacent to arrows are path coefficients, with arrow width proportional to the coefficient magnitude. R^2^ values denote the proportion of variance explained for each endogenous variable.

Path analysis (PLS-PM) was used to explore potential relationships among warming, nitrogen addition, DOM, microbial traits, and CME (Figure 9). The model fit was good (GoF = 0.560), indicating a good overall model fit for describing the relationships among variables. Bootstrap validation (1000 replicates) confirmed the robustness of three key paths: N input → soil (β = 0.944, 95% CI [0.918, 0.958]), N input → DOM (β = 0.322, 95% CI [0.119, 0.618]), and soil → DOM (β = 0.586, 95% CI [0.295, 0.783]). Analysis of path coefficients suggested distinct association patterns among variables: nitrogen addition showed a significant positive association with DOM compositional structure (R = 0.32, *p* < 0.05). Warming, however, was associated with indirect changes in DOM composition primarily through shifts in soil physicochemical properties (R = 0.58, *p* < 0.05). Meanwhile, the warming–nitrogen interaction was linked to variation in microbial functional traits largely via DOM-mediated pathways (R = −0.88, *p* < 0.05), whereas microbial community structure itself showed no significant direct association. DOM composition exhibited a positive but non-significant association with CME in bootstrap validation (R = 0.51, 95% CI [−0.097, 0.752]), suggesting a trend rather than a definitive direct relationship. Inferred microbial functional traits were significantly associated with CME (R = −0.31, *p* < 0.05), likely reflecting shifts in inferred microbial metabolic strategies under environmental change. However, this path also showed a wide bootstrap confidence interval crossing zero (95% CI [−0.643, 0.338]), indicating less robust statistical support. In addition, microbial functional traits appeared to partially mediate the relationship between DOM and CME, though bootstrap results for these indirect pathways were not statistically robust. Collectively, these results suggest that environmental drivers are associated with variation in CME largely through their relationships with microbial functional traits, with DOM acting as a key substrate-mediated pathway influencing carbon cycle processes. Several path estimates, however, exhibited reduced statistical certainty under bootstrap validation, warranting cautious interpretation.

## 4. Discussion

### 4.1. Response of Peat Soil CO_2_ Emissions and CME to Warming and Nitrogen Interaction

Our results indicate that while nitrogen addition consistently stimulated CO_2_ emissions across the three investigated peat soils, this positive effect was significantly suppressed under warming conditions (Figure 2). This points to an antagonistic interaction between nitrogen input and elevated temperature, which exerts directionally consistent controls on peat carbon turnover. Accordingly, exogenous nitrogen likely enhances microbial growth efficiency and metabolism, accelerating the decomposition of soluble organic substrates [67,68]. Nitrogen addition may also alleviate soil C:N stoichiometric imbalance, thereby stimulating microbial carbon-acquiring enzyme activity and enhancing microbial-mediated organic carbon turnover [69,70]. However, this effect was diminished under the 20 °C warming treatment (Figure 2), indicating that warming-induced changes in substrate bioavailability dampened the response of carbon turnover to nitrogen input [71]. Warming generally promotes the transformation of DOM toward more humified and recalcitrant forms, while accelerating the preferential microbial consumption of labile components. This shift reduces the overall bioavailability of the remaining substrate pool. Such patterns align with the observed changes in DOM compositional structure in this study (Figure 6b), suggesting that warming constrains the nitrogen-induced stimulation of microbial carbon decomposition by altering microbial substrate availability and functional responses through changes in DOM composition. Taken together, nitrogen addition substantially stimulated CO_2_ emissions under lower temperature conditions. However, under future warming scenarios, the sensitivity of peat organic carbon decomposition to nitrogen input is likely to diminish.

The response of CME further underscores the complexity of interactive effects in regulating peat carbon cycling. In the C and LW peat soils, warming enhanced CME under nitrogen-limited conditions, whereas under high nitrogen input, warming significantly suppressed CME (Figure 3). This shift from stimulation to inhibition highlights a context-dependent regulatory mechanism driven by warming–nitrogen coupling. Such a transition likely stems from shifts in microbial metabolic strategies driven by changes in DOM quality under warming and nitrogen enrichment. Specifically, nitrogen addition increased the proportion of humic-like components (CP2 and CP3) at the expense of protein-like, autochthonous components (CP4 and CP5) (Figure 6b). This shift toward more recalcitrant substrates may lower microbial carbon use efficiency by reducing substrate lability and elevating decomposition costs, ultimately suppressing CME under combined warming and high nitrogen [72,73]. By accelerating the shift toward more recalcitrant DOM fractions, warming may exacerbate substrate limitation under high nitrogen conditions, ultimately suppressing CME through constraints on microbial carbon utilization and metabolic efficiency [71].

In contrast to the C and LW peat soils, where CME varied consistently with nitrogen input intensity across temperature treatments, CME in the G peat soil remained largely insensitive to both warming and nitrogen addition. This pattern likely stems from the inherently higher C:N ratio and substrate recalcitrance in the G bog [74], where carbon mineralization is more strongly constrained by substrate quality. These findings suggest that microbial responses to substrate characteristics exert primary control over the strength of warming and nitrogen addition, modulating the sensitivity of carbon mineralization processes to environmental change [75,76]. The contrasting responses of CME among the three peatland vegetation types likely reflect inherent differences in substrate quality, nutrient stoichiometry, microbial adaptation, and oxygen availability. Sedge and reed peat soils generally contain relatively labile organic substrates and lower C:N ratios, which can support more active microbial metabolism and greater responsiveness to external nitrogen inputs. In contrast, shrub peat soils are often characterized by higher C:N ratios and greater proportions of recalcitrant compounds, such as lignin-derived organic matter, which may constrain microbial decomposition and reduce sensitivity to warming and nitrogen enrichment [77]. In addition, differences in vegetation-derived litter inputs can shape distinct microbial community compositions and functional potentials [78], leading to varying capacities for carbon utilization among peat types [67,79]. Shrub-dominated peat may harbor microbial communities adapted to more recalcitrant substrates, resulting in slower responses to short-term environmental changes. Furthermore, differences in peat structure among vegetation types may influence oxygen diffusion and redox conditions, thereby affecting microbial activity and carbon mineralization processes [80]. Together, these factors likely explain why shrub peat exhibited weaker CME responses than sedge and reed peat under warming and nitrogen addition.

### 4.2. Changes in DOM Compositional Structure Under Warming and Nitrogen Interactions

By excluding external DOC inputs, our incubation system ensured that DOM dynamics were driven primarily by microbial metabolic transformation of internal soil substrates. Under this framework, high nitrogen addition significantly increased both DOC concentration and the DOC/SOC ratio (Figure 4), suggesting that exogenous nitrogen promoted microbial-mediated solubilization of organic components from SOM. This observation is consistent with established evidence that nitrogen enrichment stimulates hydrolase activities targeting carbon and nitrogen, thus promoting soluble organic matter production [81,82]. In this root-free system, the nitrogen-induced DOC increase further supports the view that enhanced microbial SOM decomposition drove the mobilization of dissolved organic fractions [83].

Optical indices further evidenced the coordinated shifts in DOM chemistry driven by warming and nitrogen interactions. The observed decrease in BIX and increase in HIX under nitrogen addition (Figure 5) point to a preferential consumption of labile DOM and an accumulation of humified aromatic fractions. This implies that nitrogen enrichment accelerated the turnover of bioavailable substrates, thereby enriching the residual DOM pool in recalcitrant compounds [27,52]. The sustained decline in FI value pointed to a change in DOM provenance, indicating shifts in microbial-derived DOM inputs and an increasing dominance of more recalcitrant, terrestrially derived compounds and an enrichment of more recalcitrant, terrestrially derived compounds. This shift likely reflects nitrogen-associated changes in microbial functional traits and substrate utilization strategies, consequently reshaping the partitioning between labile and recalcitrant carbon pools [84]. Warming exerted a synergistic regulatory effect on these optical properties. The more convergent pattern observed under 20 °C indicates accelerated DOM turnover and enhanced transformation of organic matter. Elevated temperature likely stimulated microbial metabolism, enabling the utilization and degradation of previously recalcitrant substrates. This finding corroborates previous reports that warming accelerates DOM turnover, potentially exacerbating carbon losses from peatland ecosystems [85,86].

The PARAFAC analysis further corroborated the trends indicated by the optical indices. With increasing nitrogen input, the relative abundance of fulvic- and humic-like components (CP2 and CP3) increased, whereas protein-like and microbial metabolite–derived components (CP4 and CP5) declined. From the perspective of microbial substrate availability, this compositional shift implies a reduction in readily accessible carbon sources and an increasing dominance of more complex and energetically costly substrates [63]. Protein-like components (CP5), typically associated with freshly produced, labile organic matter, are rapidly utilized by microorganisms and therefore represent a key energy source for microbial metabolism. Their decline suggests a depletion of easily degradable substrates. In contrast, humic-like components (CP2 and CP3) are structurally complex and relatively recalcitrant [62], requiring specialized metabolic pathways for degradation, which may increase the metabolic cost for microorganisms. Quinone-like components (CP4), characterized by redox-active functional groups, may influence microbial processes by participating in electron transfer reactions, thereby altering microbial metabolic pathways and carbon transformation processes. This shift from labile, bioavailable fractions toward more humified and aromatic compounds suggests that nitrogen enrichment altered microbial substrate preferences and selective utilization patterns. The preferential consumption of protein-like components bearing quinone and semiquinone moieties under nitrogen enrichment may be linked to their redox-active nature [87]. These compounds, capable of reversible electron transfer, can participate in redox cycling that promotes organic matter oxidation [64,88]. Such processes likely enhance oxidative decomposition and could partially explain the elevated CO_2_ emissions observed in this study. Collectively, these changes in DOM composition are likely associated with shifts in the balance between labile and recalcitrant substrates, thereby influencing microbial carbon utilization strategies and ultimately being linked to variation in CME. Notably, CP1 (terrestrial humic-like component) showed no significant variation across treatments yet ranked among the top predictors of CME in the Random Forest model (Figure 8). This can be explained by the inherent differences in humification status among vegetation types, and individual replicates provided sufficient variation to make CP1 a meaningful predictor of baseline carbon mineralization potential. This underscores the value of incorporating DOM quality metrics—even those that are relatively stable when assessing carbon cycling responses to environmental change.

In contrast to the unidirectional influence of nitrogen enrichment, warming exerted a buffering effect on DOM composition in this study. Specifically, warming attenuated the accumulation of humic-like components (CP2 and CP3) while simultaneously slowing the decline of protein-like fractions (CP4 and CP5). This buffering pattern does not contradict the accelerated humification described in Section 4.1; rather, warming likely promotes both the formation of humic substances from labile precursors and their subsequent transformation, with the net effect of reducing proportional shifts among DOM fractions. Integrating these findings with the optical indices and PARAFAC results, warming appears to have accelerated microbial-mediated bulk metabolic turnover of DOM rather than selectively targeting specific fractions, thereby manifesting as an apparent buffering effect [89].

### 4.3. Responses of Microbial Structure and Function to Warming and Nitrogen Interactions

In the present study, the three peatland soil types (C, LW, and G) exhibited overall stability in bacterial community composition at the phylum level following warming and nitrogen addition. Except for a significant decline in the relative abundance of Bacteroidota under nutrient enrichment, the dominant phyla showed only minor compositional changes, although NMDS detected significant community separation by temperature. In contrast, functional guilds exhibited stronger and more dynamic responses to interactive effects. The contrasting responses of taxonomic structure and functional traits. reflects a decoupling between microbial taxonomic composition and functional traits. This pattern is consistent with functional–taxonomic decoupling, wherein nitrogen addition can alter microbial metabolic potential without fundamentally restructuring community composition. Such a pattern aligns with observations from diverse ecosystems, including forests, grasslands, and permafrost peatland, where climate and nutrient perturbations consistently exert stronger impacts on metabolic potential and functional attributes than on taxonomic structure [90,91,92,93].

The relatively uniform responses of functional guilds to warming and nitrogen addition underscore the coupled regulation of substrate availability and redox conditions. In this study, low nitrogen addition increased the relative abundance of carbon cycling-related functional groups (*chemoheterotrophy* and *aerobic_chemoheterotrophy*), suggesting that moderate nitrogen input enhanced microbial access to and decomposition of bioavailable substrates. Conversely, warming either reduced the abundance of nitrogen-cycling (*ureolysis*, *nitrate/nitrogen respiration*) and methane-cycling (*methanotrophy*, *methylotrophy*, *hydrocarbon degradation*) functional groups or dampened their responses to nitrogen addition. This suppression likely stems from warming-induced changes in microbial metabolic activity, including accelerated substrate turnover and increased oxygen consumption, which elevates metabolic costs and shifts terminal electron acceptor availability. These functional shifts align with a mechanism in which substrate quality and redox conditions collectively shape microbial niches. By enhancing microbial-mediated DOM humification and depleting labile carbon, while concurrently accelerating respiration and oxygen consumption, warming likely fostered localized hypoxic or anaerobic microsites. This reduction in electron acceptor availability would in turn suppress aerobic and facultatively aerobic guilds involved in nitrogen and methane cycling. These observations are supported by previous studies showing that warming alters nitrogen cycling and methane oxidation via redox modifications, while nitrogen inputs can suppress methane oxidation through ammonium competition for methane monooxygenase [94,95].

This functional reorganization carries significant implications for peat carbon stability. Enhanced heterotrophic functional traits may accelerate labile DOM and SOM mineralization, boosting CO_2_ emissions. Concurrently, suppressed nitrogen- and methane-cycling guilds could diminish nitrification, denitrification, and methane oxidation. Under sub-anaerobic conditions, these shifts may promote methane production while curtailing its consumption, thereby reshaping greenhouse gas fluxes and CME. These results caution against relying solely on taxonomic metrics when assessing microbial contributions to carbon cycling. Rather, functional traits, which emerge as key regulators of peatland carbon dynamics under warming and nitrogen interactions, warrant greater attention.

Overall, the interactive effects of warming and nitrogen addition were more strongly associated with variation in microbial functional traits than with taxonomic community composition. The responses of functional guilds were closely linked to substrate availability, shifts in redox conditions, and changes in microbial ecological niches. This microbial functional reassembly governed not only the degradation pathways and utilization efficiency of DOM but also ultimately shaped CME across the three peat soils. Collectively, these insights advance our understanding of how peatland carbon cycling may respond to the dual stressors of warming and nitrogen deposition. It should be noted that this study focused exclusively on bacterial communities due to the use of bacterial-specific 16S rRNA gene primers. Fungi and archaea also play important roles in peatland carbon cycling—fungi contribute to the degradation of recalcitrant organic matter, while archaea are key regulators of CH_4_ fluxes—and may respond to warming and nitrogen addition. Future studies incorporating ITS sequencing for fungi, archaea-specific 16S rRNA gene primers, or shotgun metagenomic approaches, together with co-occurrence network analysis with larger sample sizes, would provide a more comprehensive understanding of microbial contributions to peatland carbon dynamics and how microbial interaction networks are altered under environmental change.

### 4.4. Mechanistic Pathways Linking DOM Composition and Microbial Functional Traits to CME Under Warming and Nitrogen Interactions

In this study, we integrated Pearson correlation analysis, Random Forest modeling, and PLS-PM to systematically identify the key drivers regulating CME in peat soils. Based on these approaches, we constructed an “Environment—DOM—Microbial Function—CME” pathway framework (Figure 9) to disentangle the hierarchical regulatory mechanisms. Our integrative analysis revealed that DOM composition showed a positive association with CME and was also related to microbial functional traits, suggesting a potential intermediary role in carbon mineralization processes. These inferred functional profiles were significantly associated with CME and appeared to play a key role within this pathway.

Overall, nitrogen addition significantly increased DOC and enhanced humic-like components (CP2), whereas both warming and nitrogen addition altered DOM composition, primarily by increasing humification and structural complexity, consistent with previous studies [96,97]. Critically, PLS-PM analysis suggested that warming and nitrogen addition were associated with variation in microbial functional traits, with DOM acting as an important substrate-mediated pathway, whereas the contribution of taxonomic structure was negligible. This finding aligns with the concept of “taxonomy—function decoupling,” wherein microbial function responds more sensitively to environmental change than taxonomic composition.

When DOM quality shifts, microbial functions do not change randomly; instead, they undergo selective reorganization through a “substrate filtering effect.” In this study, *hydrocarbon_degradation*, *ureolysis*, *nitrogen_respiration*, and *methanotrophy* were identified as key regulatory functional groups. Although *methanotrophy* is primarily associated with CH_4_ oxidation, its negative correlation with CME may reflect broader shifts in carbon allocation within microbial communities. *Methanotrophs* can also contribute to heterotrophic CO_2_ production via non-methane substrates, and CO_2_ from methane oxidation contributes to total headspace CO_2_. The Random Forest model therefore captures it as an indicator of shifting carbon allocation patterns rather than as a direct estimator of methane oxidation rates. The association between DOM and CME appears to be mediated by microbial functional pathways, which may shift carbon flow toward alternative metabolic routes and thereby regulate CME. This process could reduce the direct association between DOM and CME, resulting in an apparent negative mediating effect.

This study suggests that microbial functional traits are closely associated with variation in CME under warming and nitrogen addition, with DOM acting as a key substrate-related factor through a dual role: as a source of substrates and as a filter influencing the relative importance of specific microbial functional guilds. The observed mediation patterns indicate that predictions of peatland carbon feedbacks may benefit from integrating DOM structural properties with microbial functional characteristics, rather than relying solely on DOC concentration or taxonomic composition. It should be noted that FAPROTAX-based functional annotations reflect potential metabolic capacities inferred from taxonomic composition rather than directly measured gene expression or process rates. Therefore, these results should be interpreted with caution. Future research combining metagenomic or metatranscriptomic approaches with molecular-level DOM characterization would provide more direct evidence for the proposed pathways and help validate predictions of peatland carbon dynamics under warming and nitrogen deposition.

In summary, the combined effects of warming and nitrogen addition were associated with a hierarchical cascade: altered DOM composition corresponded with shifts in inferred microbial functional traits, and these shifts were linked to variation in CME. Within this framework, such traits were identified as key factors associated with CME, while DOM served as a substrate-mediated pathway statistically linking environmental change to potential microbial metabolic processes. It should be noted that the relationships identified here are based on statistical associations derived from Random Forest and PLS-PM analyses and therefore do not establish direct causal mechanisms. Accordingly, the proposed pathways should be interpreted as hypothesis-generating frameworks rather than as definitive mechanistic relationships. These findings not only deepen our understanding of peatland carbon cycle complexity but also emphasize that future predictions of soil carbon stability under global change must jointly consider the synergistic interplay between DOM quality and microbial functional characteristics.

## 5. Conclusions

Using a controlled warming and nitrogen addition experiment, this study simulated future environmental scenarios facing peatlands and explored how environmental change is associated with soil CME. The results suggest a pathway in which warming–nitrogen interactions are linked to variation in CME largely through their associations with microbial functional traits, with DOM acting as a substrate-mediated link between environmental drivers and microbial processes. Our findings indicate that shifts in DOM composition are associated with variation in CME, potentially through corresponding changes in inferred functional profiles. Notably, high nitrogen inputs may be linked to changes in microbial metabolic pathways associated with labile carbon utilization, suggesting a potential increase in peat carbon vulnerability. These results imply that while warming may stimulate CO_2_ emissions from peatlands under low nitrogen availability, this stimulatory effect can be reversed under high nitrogen inputs, where warming reduces carbon mineralization. This suggests that the net response of peatland carbon emissions to future environmental change will depend critically on the balance between warming magnitude and local nitrogen deposition levels. Overall, this study highlights the importance of both DOM composition and inferred microbial functional traits in understanding peatland carbon dynamics under environmental change.

## Figures and Tables

**Table 1:** Excitation and emission fluorescence maxima of the five components identified by the PARAFAC model and compared with previously identified fluorescent regions.

Component	Peak Ex	Peak Em	Description	Reference
CP1	310	430	Terrestrial humic-like substance	[56,57]
CP2	270&370	480	Humic acid-type components	[58]
CP3	300&450	540	Soil fulvic acid and terrestrial, semiquinone-like components.	[59]
CP4	<250	490	Oxidized quinones	[60]
CP5	275	330	Protein-like component	[61]

## Data Availability

Data will be made available on request.

## Supplementary Materials

The following supporting information can be downloaded at: https://www.mdpi.com/article/10.3390/microorganisms14061190/s1, Figure S1: Dominant bacterial phyla in peat soils from (a) sedge, (b) reed, and (c) shrub peatlands under different incubation conditions. Figure S2: (a) Changes in bacterial α-diversity under different incubation conditions. (b1) NMDS analysis of bacterial communities in soils from the three vegetation types, and (b2–b4) in each peatland soil type (sedge, reed, and shrub) under different incubation conditions. Figure S3: Genus-level bacterial community composition in peat soils under warming and nitrogen addition. Relative abundance of the top 10 genera in (a) sedge (C), (b) reed (LW), and (c) shrub (G) peat soils. Figure S4: LEfSe-identified biomarker taxa in (a) sedge, (b) reed, and (c) shrub peat soils under factorial warming and nitrogen addition treatments (LDA > 4, *p* < 0.05). Table S1: Background values of soil physicochemical properties for different soil types. Table S2: Background values of DOM composition for different soil types. Table S3: Effect of different incubation conditions on the soil properties. Table S4: Physical properties of peat soils under different vegetation types.
