# Supplementary material for: Dissolved Organic Matter Composition and Microbial Functional Traits Regulate Carbon Mineralization Efficiency in Peatland Soils Under Experimental Warming and Nutrient Input
Source: Microorganisms. 2026 May 25;14(6):1190. doi: 10.3390/microorganisms14061190 (PMC13304410; doi:10.3390/microorganisms14061190)
Supplement: Supplementary file 1 [file microorganisms-14-01190-s001.zip › microorganisms-4273158-supplementary.pdf]

DOM Composition and Microbial Functional Traits Drive CME in Peatland soils under Experimental Warming and Nutrients Input

Yixinfei Lin[1], Hongfeng Bian[1,*], Yanan Liu[1], Pengchen Zhou[1], Xue Wang[1]

State Environmental Protection Key Laboratory of Wetland Ecology and Vegetation Restoration, School of Environment, Northeast Normal University, Changchun, Jilin 130117, China

* Correspondence: Correspondence: bianhf108@nenu.edu.cn

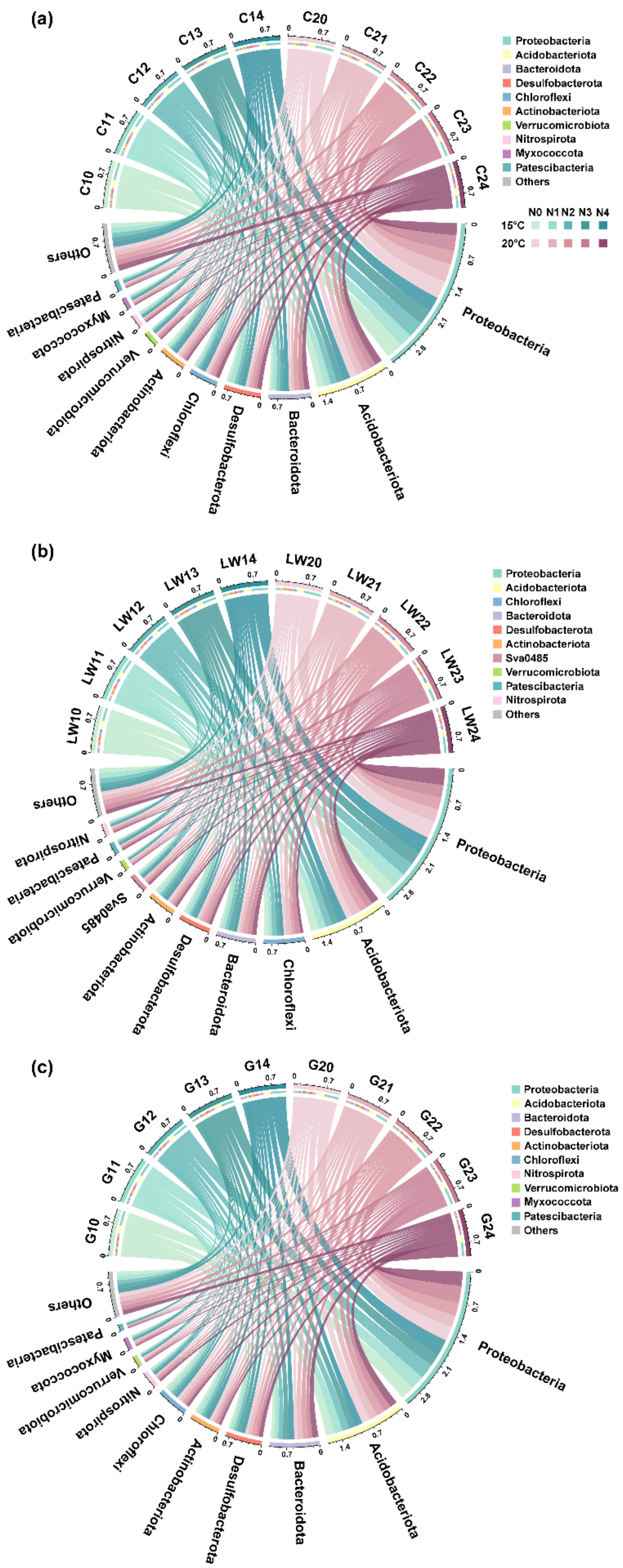

Figure S1. Dominant bacterial phyla in peat soils from (a) sedge, (b) reed, and (c) shrub peatlands under different incubation conditions.

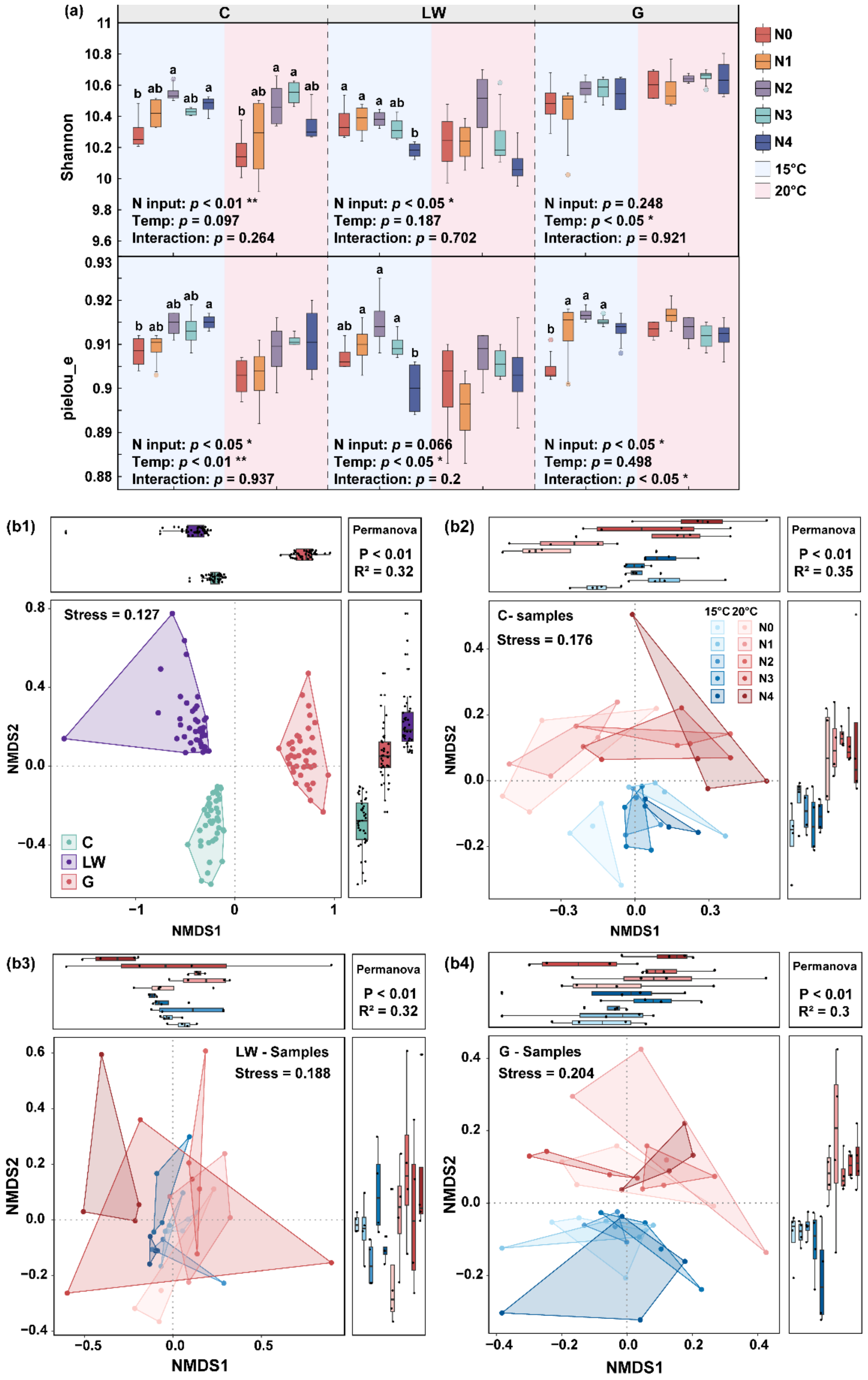

Figure S2. (a) Changes in bacterial α-diversity under different incubation conditions. (b1) NMDS analysis of bacterial communities in soils from the three vegetation types, and (b2–b4) in each peatland soil type (sedge, reed, and shrub) under different incubation conditions. Boxplot lines indicate medians with 95% confidence intervals; Different lowercase letters indicated significant differences under different N input at $P < 0.05$; $*$ represented P <0.05, $**$ represented $P < 0.01$.

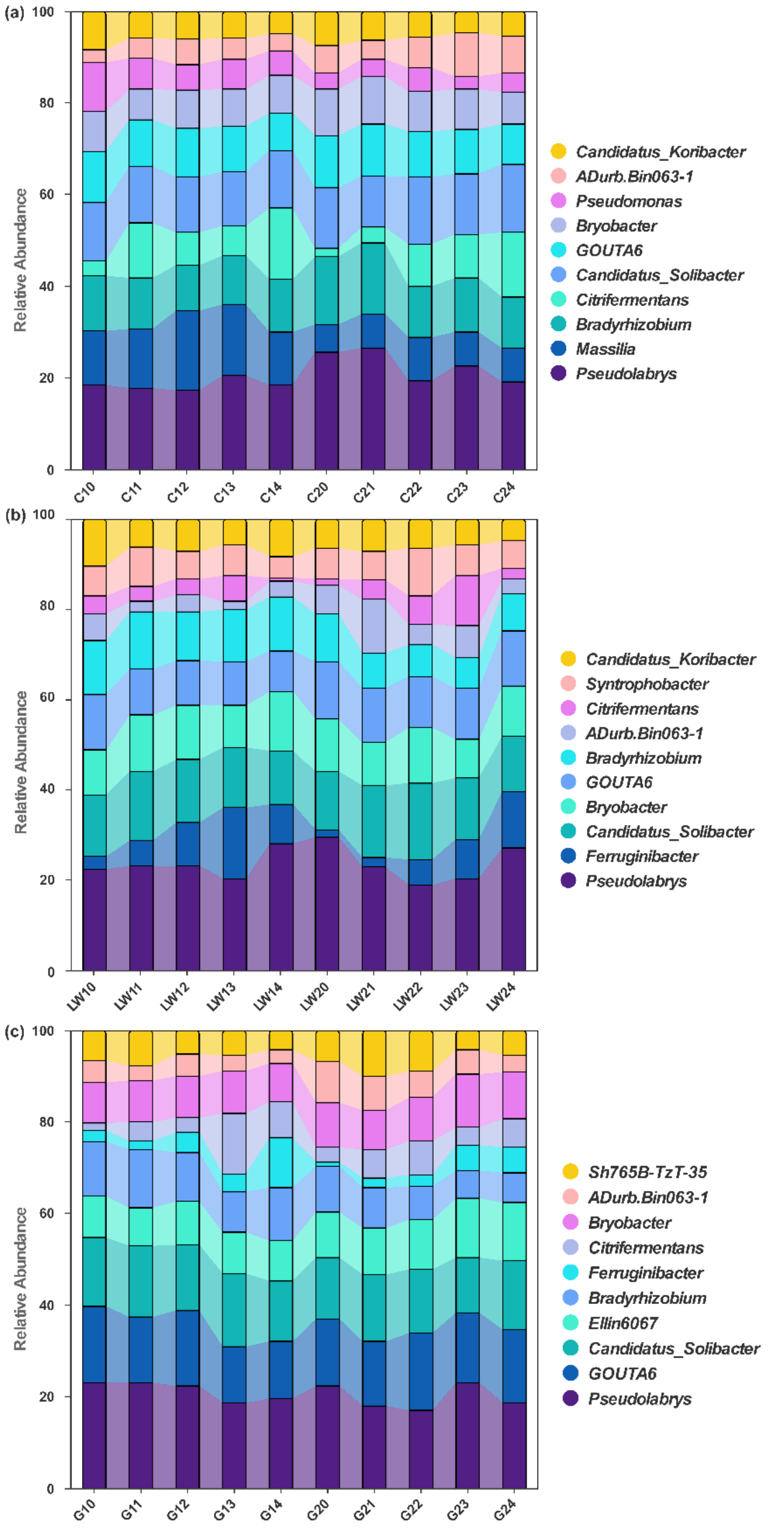

Figure S3. Genus-level bacterial community composition in peat soils under warming and nitrogen addition. Relative abundance of the top 10 genera in (a) sedge (C), (b) reed (LW), and (c) shrub (G) peat soils.

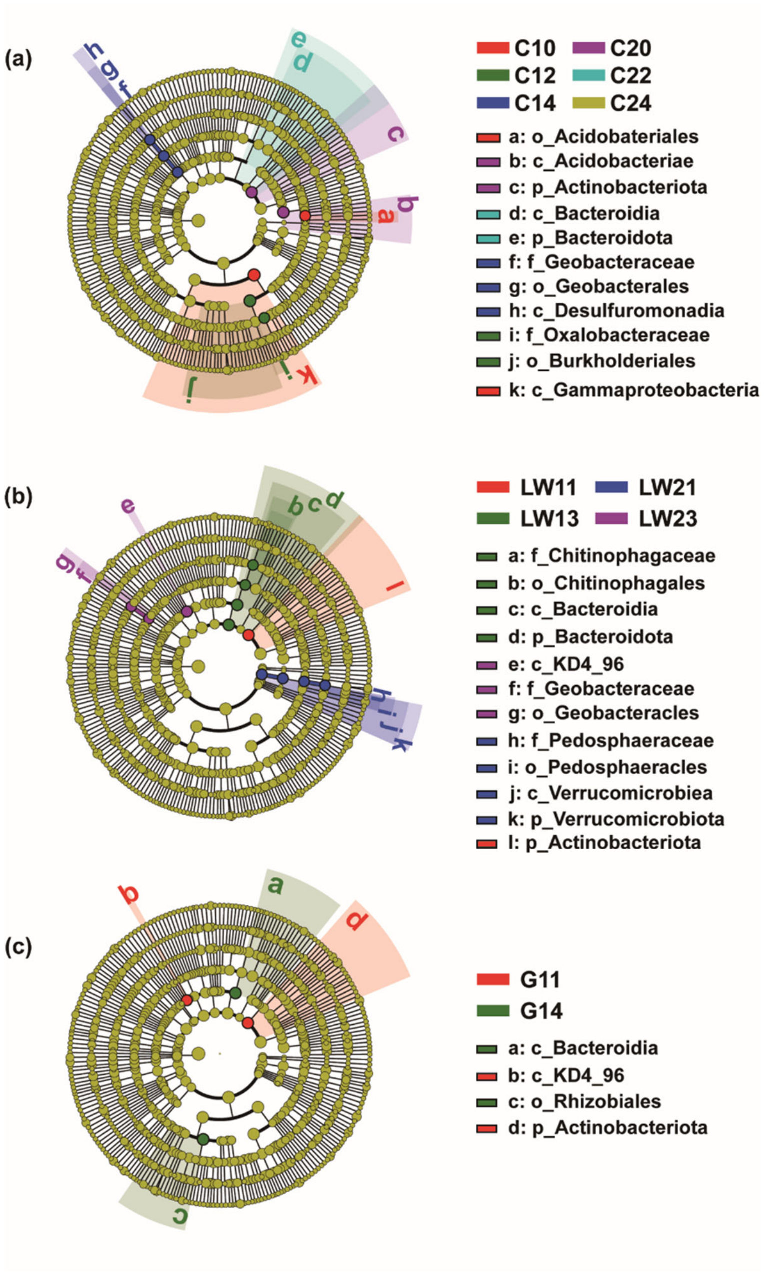

Figure S4. LEfSe-identified biomarker taxa in (a) sedge, (b) reed, and (c) shrub peat soils under factorial warming and nitrogen addition treatments (LDA > 4, $P < 0.05$).

Table S1. Background values of soil physicochemical properties for different soil types.

	C	LW	G
**DOC (mg/g)**	0.85 ± 0.02 [ab]	0.82 ± 0.03 [b]	0.97 ± 0.05 [a]
**SOC (g/kg)**	187.99 ± 5.73 [a]	182.87 ± 5.81 [ab]	167.75 ± 4.54 [b]
**DOC/SOC**	4.56 ± 0.26 [b]	4.53 ± 0.03 [b]	5.81 ± 0.27 [a]
**C/N**	14.72 ± 0.26 [b]	14.88 ± 0.08 [b]	16.12 ± 0.09 [a]

Values are expressed as mean ± SEM, n = 4. Different lowercase letters indicated significant differences for different soil types at $P < 0.05$.

Table S2. Background values of DOM composition for different soil types.

	C	LW	G
**DOM.CP1%**	37.61 ± 0.29 ^{b}	37.71 ± 0.25 ^{b}	39.57 ± 0.36 ^{a}
**DOM.CP2%**	20.16 ± 0.18 ^{b}	20.72 ± 0.14 ^{b}	25.28 ± 0.51 ^{a}
**DOM.CP3%**	6.73 ± 0.10 ^{c}	7.55 ± 0.06 ^{b}	8.51 ± 0.31 ^{a}
**DOM.CP4%**	13.25 ± 0.50 ^{a}	13.44 ± 0.26 ^{a}	10.40 ± 0.55 ^{b}
**DOM.CP5%**	22.24 ± 0.30 ^{a}	20.57 ± 0.66 ^{a}	16.24 ± 0.69 ^{b}

Values are expressed as mean ± SEM, n = 4. Different lowercase letters indicated significant differences for different soil types at $P < 0.05$.

Table S3. Effect of different incubation conditions on the soil properties.

		C					LW					G				
		**N0**	**N1**	**N2**	**N3**	**N4**	**N0**	**N1**	**N2**	**N3**	**N4**	**N0**	**N1**	**N2**	**N3**	**N4**
**pH**	**15°C**	4.75 ± 0.06 ^{c}	5.05 ± 0.01 ^{abc}	5.25 ± 0.01 ^{abc}	5.55 ± 0.01 ^{ab}	5.74 ± 0.01 ^{a}	5.13 ± 0.01 ^{e}	5.43 ± 0.01 ^{d}	5.49 ± 0.01 ^{c}	5.73 ± 0.01 ^{b}	5.78 ± 0.00 ^{a}	5.02 ± 0.02 ^{e}	5.15 ± 0.01 ^{d}	5.32 ± 0.01 ^{c}	5.42 ± 0.01 ^{b}	5.54 ± 0.01 ^{a}
	**20°C**	4.96 ± 0.04 ^{e}	5.08 ± 0.01 ^{d}	5.33 ± 0.01 ^{c}	5.46 ± 0.01 ^{b}	5.71 ± 0.02 ^{a}	5.14 ± 0.01 ^{c}	5.23 ± 0.00 ^{bc}	5.40 ± 0.00 ^{abc}	5.54 ± 0.01 ^{ab}	5.66 ± 0.00 ^{a}	5.08 ± 0.01 ^{d}	5.12 ± 0.00 ^{c}	5.21 ± 0.01 ^{b}	5.35 ± 0.01 ^{a}	5.35 ± 0.01 ^{a}
**EC**	**15°C**	200.93 ± 14.88 ^{b}	219.75 ± 5.44 ^{ab}	229.50 ± 7.37 ^{ab}	232.50 ± 5.52 ^{ab}	264.75 ± 4.92 ^{a}	210.75 ± 2.17 ^{c}	210.50 ± 1.32 ^{c}	266.25 ± 5.47 ^{b}	270.00 ± 3.44 ^{b}	324.25 ± 3.57 ^{a}	197.00 ± 1.35 ^{e}	216.75 ± 3.57 ^{d}	236.25 ± 2.69 ^{c}	249.25 ± 2.46 ^{b}	275.00 ± 2.52 ^{a}
	**20°C**	187.78 ± 7.26 ^{b}	208.25 ± 2.87 ^{ab}	205.75 ± 3.66 ^{ab}	230.75 ± 7.89 ^{a}	240.25 ± 1.93 ^{a}	196.55 ± 2.33 ^{d}	231.75 ± 4.50 ^{c}	227.00 ± 4.12 ^{c}	256.75 ± 2.43 ^{b}	304.00 ± 3.70 ^{a}	182.75 ± 1.70 ^{c}	213.25 ± 2.32 ^{bc}	224.00 ± 0.41 ^{abc}	246.00 ± 2.08 ^{ab}	283.50 ± 10.27 ^{a}
**NH_4^+-N (mg N / kg soil)**	**15°C**	19.9 ± 0.36 ^{b}	45.00 ± 1.40 ^{b}	84.50 ± 5.76 ^{ab}	110.53 ± 0.99 ^{a}	148.01 ± 8.97 ^{a}	21.70 ± 1.22 ^{c}	47.21 ± 3.30 ^{bc}	78.23 ± 5.78 ^{abc}	101.49 ± 1.90 ^{ab}	135.00 ± 5.21 ^{a}	15.31 ± 1.14 ^{d}	37.90 ± 2.92 ^{c}	74.57 ± 3.85 ^{b}	85.33 ± 4.41 ^{b}	125.35 ± 5.21 ^{a}
	**20°C**	20.83 ± 0.77 ^{e}	47.35 ± 0.88 ^{d}	66.42 ± 3.53 ^{c}	96.21 ± 1.00 ^{b}	120.79 ± 3.97 ^{a}	19.85 ± 0.90 ^{d}	40.68 ± 2.41 ^{c}	62.71 ± 7.66 ^{b}	69.75 ± 3.33 ^{b}	108.56 ± 2.76 ^{a}	12.35 ± 1.15 ^{e}	27.79 ± 0.61 ^{d}	46.10 ± 2.16 ^{c}	68.10 ± 1.19 ^{b}	85.87 ± 6.35 ^{a}
**NO_3^--N (mg N / kg soil)**	**15°C**	3.10 ± 0.72 ^{a}	5.19 ± 0.87 ^{a}	3.89 ± 1.14 ^{a}	4.41 ± 1.33 ^{a}	2.68 ± 1.39 ^{a}	3.86 ± 1.18 ^{a}	4.41 ± 0.82 ^{a}	5.56 ± 1.38 ^{a}	5.38 ± 0.63 ^{a}	7.14 ± 1.14 ^{a}	1.49 ± 0.72 ^{b}	2.64 ± 0.78 ^{ab}	4.95 ± 0.79 ^{a}	5.92 ± 0.84 ^{a}	5.55 ± 1.54 ^{a}
	**20°C**	5.15 ± 1.79 ^{a}	5.31 ± 0.61 ^{a}	5.77 ± 1.14 ^{a}	5.40 ± 1.55 ^{a}	4.62 ± 1.63 ^{a}	5.14 ± 0.64 ^{b}	6.43 ± 0.50 ^{b}	10.34 ± 2.96 ^{b}	9.34 ± 1.24 ^{b}	20.48 ± 1.18 ^{a}	1.77 ± 0.61 ^{c}	2.86 ± 0.97 ^{bc}	4.70 ± 0.40 ^{abc}	5.22 ± 0.67 ^{ab}	7.90 ± 1.60 ^{a}
**DOC (mg/g)**	**15°C**	0.69 ± 0.01 ^{c}	0.68 ± 0.01 ^{c}	0.79 ± 0.03 ^{b}	0.82 ± 0.02 ^{b}	0.91 ± 0.03 ^{a}	0.53 ± 0.01 ^{b}	0.63 ± 0.01 ^{ab}	0.66 ± 0.01 ^{ab}	0.81 ± 0.03 ^{a}	0.84 ± 0.05 ^{a}	0.63 ± 0.01 ^{c}	0.67 ± 0.01 ^{bc}	0.70 ± 0.02 ^{b}	0.70 ± 0.01 ^{b}	0.86 ± 0.02 ^{a}
	**20°C**	0.72 ± 0.01 ^{d}	0.74 ± 0.01 ^{d}	0.83 ± 0.02 ^{c}	0.88 ± 0.02 ^{b}	0.96 ± 0.01 ^{a}	0.71 ± 0.02 ^{ab}	0.74 ± 0.04 ^{ab}	0.69 ± 0.02 ^{b}	0.78 ± 0.01 ^{ab}	0.94 ± 0.01 ^{a}	0.57 ± 0.02 ^{b}	0.62 ± 0.02 ^{ab}	0.71 ± 0.08 ^{ab}	0.66 ± 0.02 ^{ab}	0.73 ± 0.03 ^{a}
**DTN (mg/g)**	**15°C**	0.03 ± 0.00 ^{e}	0.05 ± 0.00 ^{d}	0.06 ± 0.00 ^{c}	0.07 ± 0.00 ^{b}	0.08 ± 0.00 ^{a}	0.03 ± 0.00 ^{c}	0.05 ± 0.00 ^{bc}	0.07 ± 0.00 ^{abc}	0.08 ± 0.00 ^{ab}	0.10 ± 0.00 ^{a}	0.03 ± 0.00 ^{e}	0.04 ± 0.00 ^{d}	0.06 ± 0.00 ^{c}	0.07 ± 0.00 ^{b}	0.09 ± 0.00 ^{a}
	**20°C**	0.04 ± 0.00 ^{e}	0.06 ± 0.00 ^{d}	0.07 ± 0.00 ^{c}	0.08 ± 0.00 ^{b}	0.09 ± 0.00 ^{a}	0.04 ± 0.00 ^{e}	0.06 ± 0.00 ^{d}	0.07 ± 0.00 ^{c}	0.08 ± 0.00 ^{b}	0.10 ± 0.00 ^{a}	0.03 ± 0.00 ^{e}	0.04 ± 0.00 ^{d}	0.05 ± 0.00 ^{c}	0.06 ± 0.00 ^{b}	0.08 ± 0.00 ^{a}
**SOC (g/kg)**	**15°C**	178.47 ± 12.62 ^{a}	205.36 ± 27.34 ^{a}	180.89 ± 33.32 ^{a}	185.29 ± 4.02 ^{a}	180.52 ± 9.99 ^{a}	189.40 ± 13.04 ^{a}	184.85 ± 10.32 ^{a}	174.33 ± 2.70 ^{a}	178.35 ± 6.52 ^{a}	175.35 ± 11.68 ^{a}	164.50 ± 6.82 ^{a}	180.95 ± 4.68 ^{a}	178.08 ± 9.19 ^{a}	173.62 ± 6.96 ^{a}	167.64 ± 15.67 ^{a}
	**20°C**	194.62 ± 4.49 ^{c}	170.55 ± 8.70 ^{abc}	186.50 ± 6.06 ^{ab}	165.46 ± 3.79 ^{bc}	166.73 ± 2.63 ^{a}	175.91 ± 2.39 ^{a}	187.32 ± 4.60 ^{ab}	172.73 ± 2.16 ^{b}	177.19 ± 4.24 ^{a}	185.55 ± 3.84 ^{ab}	169.55 ± 2.39 ^{a}	158.04 ± 11.39 ^{a}	176.08 ± 13.55 ^{a}	203.29 ± 41.34 ^{a}	174.58 ± 1.37 ^{a}

Values are expressed as mean ± SEM, n = 4. Different lowercase letters indicated significant differences at the same temperature under different N input at $P < 0.05$.

Table S4. Physical properties of peat soils under different vegetation types.

	C	LW	G
SWC (%)	86.28 ± 0.16 c	87.05 ± 0.26 b	87.88 ± 0.22 a
BD ($g \cdot cm^{-3}$)	0.70 ± 0.02 a	0.72 ± 0.01 a	0.74 ± 0.02 a

Values are expressed as mean ± SEM, n = 4.SWC, soil water content; BD, bulk density. Different lowercase letters indicated significant differences in different vegetation types at $P < 0.05$.
